# REG4 promotes the proliferation and anti-apoptosis of cancer

**DOI:** 10.3389/fcell.2022.1012193

**Published:** 2022-09-12

**Authors:** Hua-Chuan Zheng, Hang Xue, Cong-Yu Zhang

**Affiliations:** ^1^ Department of Oncology and Central Laboratory, The Affiliated Hospital of Chengde Medical University, Chengde, China; ^2^ Cancer Center, The First Affiliated Hospital of Jinzhou Medical University, Jinzhou, China

**Keywords:** cancer, REG4, tumor suppressor, tumor phenotype, transcriptional regulation

## Abstract

Regenerating islet-derived 4 (REG4) gene was discovered by high-throughput sequencing of ulcerative colitis cDNA libraries. REG4 is involved in infection and inflammation by enhancing macrophage polarization to M2, via activation of epidermal growth factor receptor (EGFR)/Akt/cAMP-responsive element binding and the killing inflammatory *Escherichia coli*, and closely linked to tumorigenesis. Its expression was transcriptionally activated by caudal type homeobox 2, GATA binding protein 6, GLI family zinc finger 1, SRY-box transcription factor 9, CD44 intracytoplasmic domain, activating transcription factor 2, and specificity protein 1, and translationally activated by miR-24. REG4 can interact with transmembrane CD44, G protein-coupled receptor 37, mannan and heparin on cancer cells. Its overexpression was observed in gastric, colorectal, pancreatic, gallbladder, ovarian and urothelial cancers, and is closely linked to their aggressive behaviors and a poor prognosis. Additionally, REG4 expression and recombinant REG4 aggravated such cellular phenotypes as tumorigenesis, proliferation, anti-apoptosis, chemoradioresistance, migration, invasion, peritoneal dissemination, tumor growth, and cancer stemness via EGFR/Akt/activator protein-1 and Akt/glycogen synthase kinase three β/β-catenin/transcription factor 4 pathways. Sorted REG4-positive deep crypt secretory cells promote organoid formation of single Lgr5 (+) colon stem cells by Notch inhibition and Wnt activation. Histologically, REG4 protein is specifically expressed in neuroendocrine tumors and signet ring cell carcinomas of the gastrointestinal tract, pancreas, ovary, and lung. It might support the histogenesis of gastric intestinal–metaplasia–globoid dysplasia–signet ring cell carcinoma. In this review, we summarized the structure, biological functions, and effects of REG4 on inflammation and cancer. We conclude that REG4 may be employed as a biomarker of tumorigenesis, subsequent progression and poor prognosis of cancer, and may be a useful target for gene therapy.

## Introduction

In 1984, Yonemura et al. (Yonemura et al., 1984) discovered regenerating islet-derived (REG) proteins during the regeneration of pancreatic islets. The REG family belongs to the calcium-dependent lectin (C-type lectin) gene superfamily, which encodes four multi-functional and secreted small proteins. REG proteins serve as anti-apoptotic factors, acute phase reactants, lectins, and growth factors for neural cells, pancreatic β cells, and epithelial cells in the digestive system. To date, researchers have identified human *REG I* (*Iα and Iβ*), *REG III* (*III* and *HIP/PAP*), and *REG4*, which encode homologous 158–175aa proteins. *REG* genes are located on chromosomes 2p12 (*HIP/PAP*, *REG Iα*, *REG Iβ*, and *REG III*) and 1q12-q21 (*REG4*) (Nata et al., 2004).

### The discovery and expression profile of REG4

**FIGURE 1:**
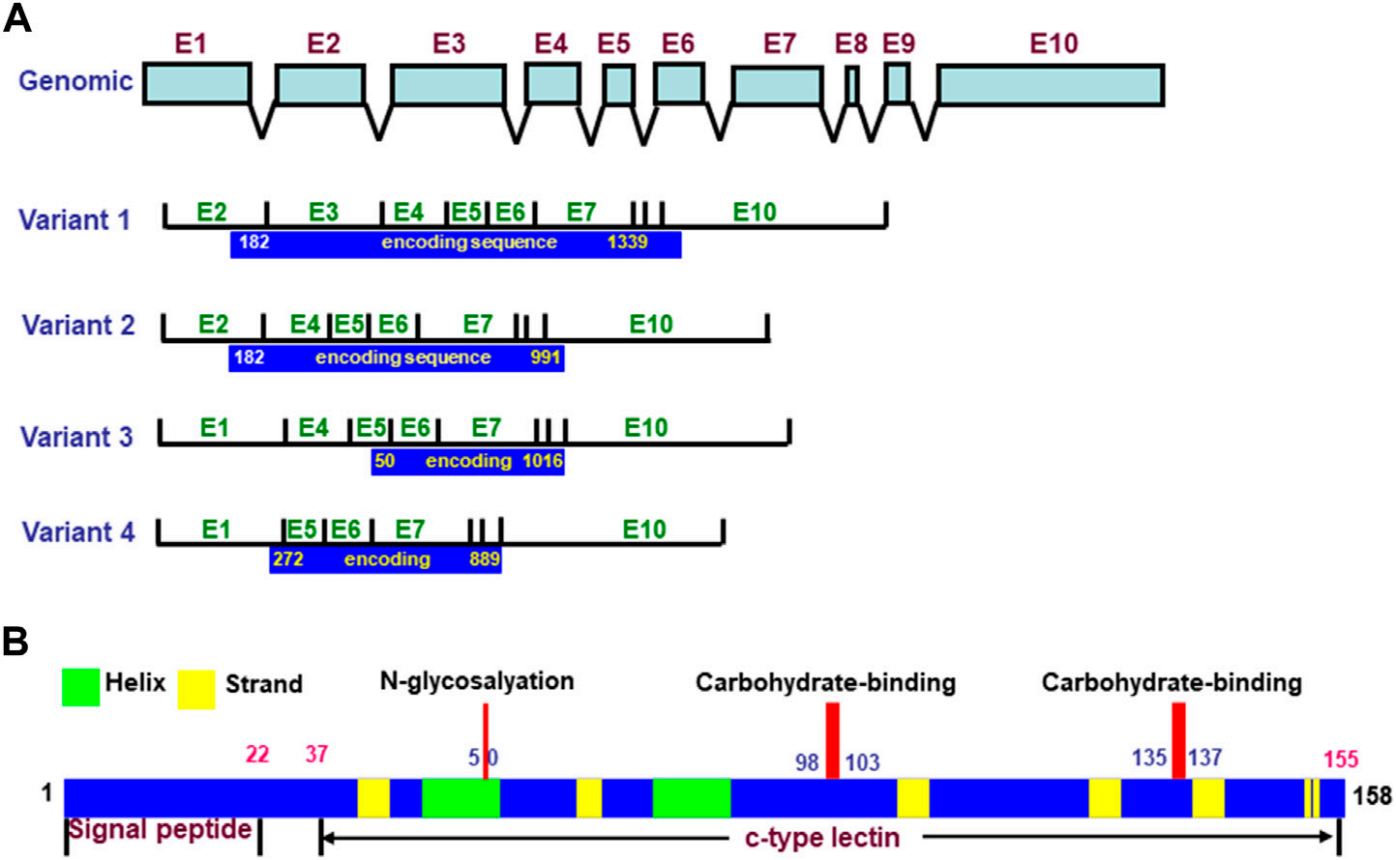
Structures of *REG4* gene and protein. The *REG4* gene has 10 exons and is alternatively spliced into four variants with different open reading frames **(A)**. The encoding protein of *REG4* variant one produces a 158aa protein with signal peptide and c-type lectin domain **(B)**.

REG4 was discovered by high-throughput sequencing of a cDNA library from an ulcerative colitis (UC) sample in 2001 (Hartupee et al., 2001). The *REG4* gene has 10 exons and encodes four types of variants by alternative splicing, which contain different open reading frames. Its longest cDNA has an open reading frame of 477 bp and encodes an 18-kDa peptide of 158aa. The REG4 protein is composed of a 22aa signal peptide and calcium-dependent lectin domain, within which are an N-glycosylation site and two carbohydrate binding sites (Figure 1) (Zhang et al., 2021). In the rat, *REG4* mRNA was detected in the brain cortex, stomach, pancreas, spleen, small intestine, colon, kidney, and urinary bladder, but not in the sciatic nerve, thymus, liver, cerebellum, suprarenal gland, heart, soleus muscle, lung, and esophagus by reverse transcriptase–PCR. Using western blot, REG4 protein expression was detectable in the pancreas, stomach, small intestine, colon, spleen, brain cortex, kidney, and urinary bladder, but not in the sciatic nerve, liver, thymus, cerebellum, suprarenal gland, soleus muscle, heart, esophagus, and lung. Immunohistochemically, positive REG4 staining was detected throughout the gastric mucosa and was mainly distributed in the basal portion of intestinal crypts. Pancreatic acinar cells appeared positive for REG4, but not pancreatic islet β cells. REG4 was expressed in large spleen cells with a large nucleus in the red pulp, but not the white pulp. Immunoreactivity for REG4 was found in large neurons of the brain cortex, and in glomerular and urinary bladder epithelial cells, but rarely in renal tubular cells (Azman et al., 2011). REG4 immunoreactivity was significantly higher in the ovary than the uterus. The expression of REG4 was strongly detectable in oocytes and granulosa cells of ovarian follicles, interstitial cells and corpus luteum, while only weak expression was found in the glandular and luminal epithelium of the rat endometrium (Du and Yao, 2013). The tissue-specific expression of REG4 is closely linked to its biological function in different organs.

### The regulation of REG4 expression

In mammalian cells, caudal type homeobox 2 (CDX2) was found to induce *REG4* expression by binding to consensus CDX2-binding elements upstream of the *REG4* gene, supported by the positive relationship between CDX2 and REG4 expression in gastric cancer cells and tissues (Naito et al., 2012; Chai et al., 2021). MicroRNA (miR)-363 suppressed the translation of GATA binding protein 6 (GATA6), which functioned as a transcriptional factor to induce REG4 and leucine-rich repeat-containing G-protein coupled receptor 5 (Lgr5) expression essential for the growth of colon cancer cells under adherent conditions (Kawasaki et al., 2015). The key transcriptional factor in the Hedgehog signaling pathway, GLI family zinc finger 1 (GLI1) bound to *REG4* promoter regions (GATCATCCA) for its transcription and translation in pancreatic cancer cells, supported by the synergic expression of REG4 and GLI1 (Wang et al., 2011). Additionally, SRY-box transcription factor 9 (SOX9) knockdown upregulated REG4 protein expression in gastric cancer cells; a positive correlation of REG4 expression with SOX9 expression in gastric cancer was noted (Zhang et al., 2018). The activating transcription factor 2 (ATF2) targeted the *REG4* promoter to induce *REG4* expression during enteritis (Xiao et al., 2019). Duan et al. (Duan et al., 2014) found that the tumor suppressor, miR-24, translationally restrained the progression of gastric cancer by down-regulating REG4. In short, *REG4* expression was transcriptionally activated by CDX2, GATA6, GLI1, ATF2, and SOX9, and translationally activated by miR-24. However, further miRNAs may be discovered in future that are associated with the translational regulation of *REG4*.

### REG4-related signal pathways

As for the cellular signaling pathway, recombinant human REG4 (rhREG4) treatment resulted in anti-apoptosis of colorectal cancer cells with the overexpression of B cell lymphoma-extra large (Bcl-xL), B cell lymphoma 2 (Bcl-2), survivin, and matrix metalloproteinase-7 (MMP-7), and the phosphorylation of epidermal growth factor receptor (EGFR) at Tyr992 and Tyr1068, and Akt at Thr308 and Ser473. It also strengthened the transcriptional activity of activator protein-1 (AP-1) by interaction with JunB, JunD, and FosB (Bishnupuri et al., 2006b). rhREG4 treatment also protected normal intestinal crypt cells from irradiation-induced apoptosis by enhancing the expression of Bcl-2, Bcl-xL, and survivin, in agreement with data from human colorectal cancer cells (Bishnupuri et al., 2010). rhREG4 treatment promoted G_2_ progression for the mitogenesis of colorectal cancer cells by Akt/glycogen synthase kinase three β (GSK3β)/β-catenin/transcription factor 4 (TCF-4) signaling (Bishnupuri et al., 2014). Meanwhile, REG4 might protect acinar cells against necrosis in experimental pancreatitis by enhancing the expression of Bcl-2 and Bcl-xL *via* activation of the EGFR/Akt pathway (Hu et al., 2011). Li et al. (Li et al., 2011) found anti-tumor effects of proteoglycan from *Phellinus linteus* on colorectal cancer cells *via* inactivation of the REG4/EGFR/Akt pathway. Taken together, these findings suggested that REG4 is a potent activator of the EGFR/Akt pathway for the proliferation and anti-apoptosis of colorectal cancer cells.

**FIGURE 2:**
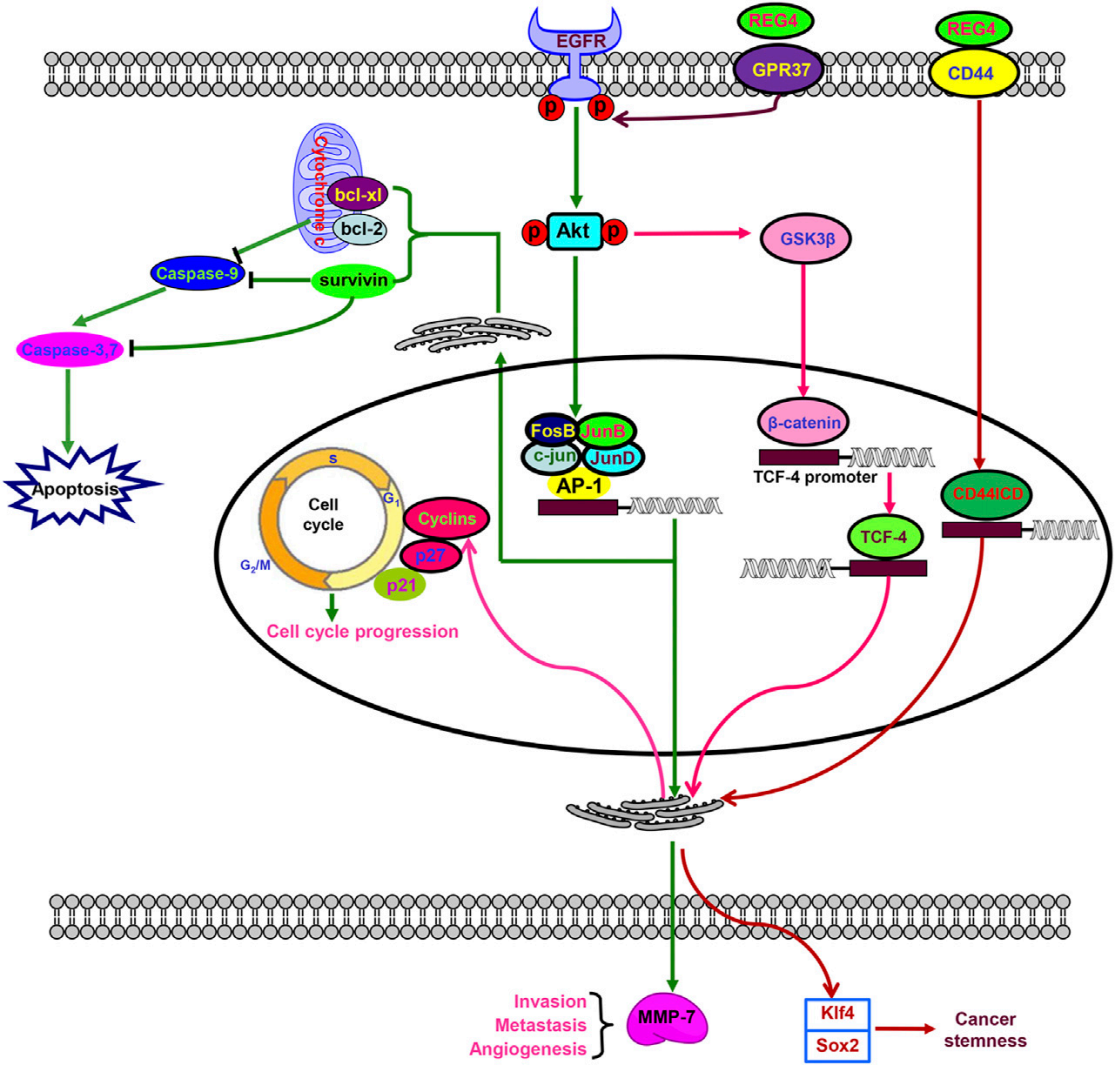
Biological functions of REG4. REG4 indirectly interacts with epidermal growth factor receptor (EGFR) to phosphorylate and activate Akt, which induces activator protein-1 (AP-1)–mediated transcriptional initiation by an AP-1/c-Jun/JunA/JunB complex and glycogen synthase kinase three β (GSK3β)–β-catenin–transcription factor 4 (TCF-4) signaling. Additionally, REG4 can bind to G protein-coupled receptor 37 (GPR37) to activate EGFR. REG4 interacts with CD44 to activate regulated transmembrane proteolysis of CD44 resulting in γ-secretase–mediated cleavage and release of the CD44 intracytoplasmic domain (CD44ICD). TCF-4 and CD44ICD serve as transcriptional activators that up-regulate the expression of cyclins for cell-cycle progression. CD44ICD-mediated Kruppel-like factor 4 (Klf4) and SRY-box transcription factor 2 (SOX2) expression is involved in cancer stemness. Activator protein-1–induced overexpression of B cell lymphoma-extra large (Bcl-xL), B cell lymphoma 2 (Bcl-2), survivin, and matrix metalloproteinase-7 (MMP7) plays an important role in apoptosis, angiogenesis, invasion, and metastasis.

In addition, Ho et al. (2010) demonstrated that REG4 bound to mannan and heparin in the absence of calcium. In addition, REG4 was found to interact with CD44 to activate its regulated intramembrane proteolysis. This resulted in the γ-secretase–mediated cleavage and release of the CD44 intracytoplasmic domain (CD44ICD) that functions as a transcriptional activator of D-type cyclins involving in cell proliferation, and Kruppel-like factor 4 and SRY-box transcription factor 2 (SOX2) expression involved in the pluripotency of cancer stem cells (Figure 2) (Bishnupuri et al., 2022). A significant correlation between REG4 and CD44 or CD44ICD supported the above-mentioned hypothesis (Sninsky et al., 2021). Liu et al. (2013) found that REG4 down-regulation also resulted in the hypoexpression of p21 and p27, which negatively regulated cyclin D1 and blocked the G_1_/S transition of prostate cancer cells. Wang et al. (2016) showed that transforming growth factor (TGF)-α stimulated specificity protein 1 (SP1) to transcriptionally promote REG4 expression, while G protein-coupled receptor 37 (GPR37) complexed with REG4, which mediated EGFR signal transduction by REG4 and promoted peritoneal metastasis of gastric cancer cells. Therefore, TGF-α/EGFR/SP1 was responsible for the transcriptional activation of REG4 in a positive feedback loop.

### Infection and inflammation

*REG4* gene was screened from UC samples (Hartupee et al., 2001). *REG4* mRNA was found to be up-regulated in Crohn’s disease and UC samples (Takasawa et al., 2018). Nanakin et al. (2007) found that *REG4* mRNA was strongly expressed in inflammatory epithelium, and dysplastic and cancerous lesions, and positively correlated with the expression of basic fibroblast growth factor (bFGF) and hepatocyte growth factor (HGF) mRNA expression in UC. In pediatric patients with intestinal failure, serum REG4 was positively correlated with serum interleukin (IL)-6 and tumor necrosis factor (TNF)-α, and REG4 protein was increased and highly expressed toward the luminal face of inflamed intestine. In intestinal conditional REG4 knockout mice, REG4 abrogation altered the colonic bacterial composition, and weakened bacterial adhesion to the colonic mucosa, finally ameliorating dextran sodium sulfate-induced colitis (Xiao et al., 2019). Further study indicated that REG4 stimulated complement-mediated attack complexes to eliminate intestinal dominant *Escherichia coli* in order to maintain homeostasis. These results supported the protective effects of REG4 on the epithelia of UC, possibly by being anti-inflammatory and anti-infection (Qi et al., 2020).

Hu et al. (Hu et al., 2011) also found that REG4 expression was significantly up-regulated during acute pancreatitis. The REG4 secreted by pancreatic cancer cells promoted macrophage polarization to M2 via EGFR/Akt/cAMP-responsive element binding activation, finally promoting tumor growth and distant metastasis (Ma et al., 2016). Li et al. (2021) found that rhREG4 attenuated the severity of rat osteoarthritis by facilitating the proliferation of articular chondrocytes. In a rat model of acutely-injured liver, treatment with recombinant interleukin 22 (IL-22) lentivirus reduced serum total bilirubin, alanine and aspartate transaminases, and enhanced REG4 expression, suggesting that REG4 might be involved in the protective effects of IL-22 on hepatic injury (Zhang et al., 2015).

### Gastric cancer

At the mRNA level, *REG4* gene was significantly up-regulated in gastric cancer compared with normal mucosa (Tao et al., 2011), while our group showed higher *REG4* expression in intestinal metaplasia than in gastritis and gastric cancer (Zheng et al., 2010). *REG4* mRNA was found to positively correlate with the wall penetration (Miyagawa et al., 2008), depth of invasion, and clinicopathological stages (Ying et al., 2013) of gastric cancer. These results indicate that *REG4* mRNA expression might reflect gastric carcinogenesis and subsequent progression.

In the stomach, foveolar epithelium was negative for REG4, whereas goblet and neuroendocrine cells of intestinal metaplasia were positive for REG4 (Oue et al., 2005). Meanwhile, REG4 expression was significantly associated with both the intestinal mucin phenotype (mucin 2 [MUC2] and CDX2) and neuroendocrine differentiation of gastric cancer (Oue et al., 2005; Yamagishi et al., 2009). Zheng et al. (2010) reported that REG4 immunostaining was gradually decreased from intestinal metaplasia, adenoma, cancer to gastritis, positively correlated with mucin 5AC (MUC-5AC) and MUC-2 expression, and was most frequently expressed in signet ring cell carcinoma (SRCCs). The expression of REG4 was found to be significantly correlated with advanced T stage, N stage, M stage, TNM stage, frequent peritoneal recurrence and dissemination, diffuse-type carcinoma, and dedifferentiation of gastric cancer (Yamagishi et al., 2009; Tao et al., 2011; Moon et al., 2012). REG4 was detected in peritoneal lavage fluids of gastric cancer patients as well (Miyagawa et al., 2008; Kuniyasu et al., 2009; Yamagishi et al., 2009). The serum REG4 level was higher in patients with gastric cancer than in healthy individuals, in advanced than early gastric cancer patients, and in pre-surgical than post-surgical gastric cancer patients respectively (Mitani et al., 2007; Miyagawa et al., 2008; Kobayashi et al., 2010; Zheng et al., 2010). As for prognosis, REG4 immunoexpression was considered as an independent prognostic factor for both worse peritoneal recurrence-free and overall survival (Miyagawa et al., 2008; Tao et al., 2011; Moon et al., 2012). These data demonstrated that REG4 expression is involved in the histogenesis of gastric SRCC, neuroendocrine differentiation, and gastric carcinogenesis, and is closely linked to aggressive behaviors and adverse prognosis in gastric cancers.

With regard to drug resistance, Ying et al. (2013) found that up-regulation of *REG4* mRNA was closely linked to the intrinsic drug resistance of gastric cancer cells to fluorouracil (5-FU) or its combination therapy. All 14 REG4-positive patients with gastric cancer showed no change or disease progression when treated with a combination of low-dose 5-FU and cisplatin (Mitani et al., 2007). In gastric cancer cells, REG4 enhanced the resistance of gastric cancer cells to 5-FU through the mitogen-activated protein kinase/extracellular-signal-regulated kinase/Bim pathway (Jin et al., 2017). REG4 antibody significantly inhibited proliferation and chemosensitivity of gastric cancer cells to 5-FU (Zhang et al., 2019) and REG4 silencing caused the loss of stemness properties (Zhou et al., 2013). It was suggested that REG4 overexpression predicted chemoresistance in gastric cancer cells, possibly by promoting proliferation and stemness.

As for molecular mechanisms, REG4 expression facilitated invasion and migration of gastric cancer cells by up-regulating SOX9 expression, in contrast to REG4 knockdown (Zhang et al., 2018). Another report described how REG4 promoted proliferation, tumor growth, and migration of gastric cancer cells through the protein kinase B pathway (Huang et al., 2014a). Katsuno et al. (2012) found that coexpression of aldehyde dehydrogenase one and REG4 was involved in the tumorigenesis of diffuse-type gastric carcinoma, which was blocked by TGF-β. Kuniyasu et al. (2009) found that REG4 overexpression increased levels of Bcl-xL, Bcl-2, survivin, phosphorylated Akt, and EGFR, and decreased nitric oxide-induced apoptosis in gastric cancer cells, in contrast to REG4 silencing. In mice models of gastric cancer, REG4 expression enhanced peritoneal metastasis, weakened apoptosis, and shortened survival time (Miyagawa et al., 2008; Kuniyasu et al., 2009). These results demonstrated that REG4 aggravated the proliferation, anti-apoptosis, tumor growth, and peritoneal metastasis of gastric cancer cells.

### Colorectal cancer

At the genetic level, Lu et al. (2013) found that a single nucleotide polymorphism in *REG4* might be a genetic marker for the progression of colorectal cancer. In colorectal tissues, *REG4* mRNA–positive cells are mostly enteroendocrine and goblet cells. Adenomatous and cancer cells positive for *REG4* mRNA exhibited enterocyte-like, mucus-secreting, or undifferentiated features (Violette et al., 2003), in agreement with observations in the stomach (Oue et al., 2005; Yamagishi et al., 2009; Zheng et al., 2010). *REG4* mRNA was found to be highly expressed in all adenoma samples, with or without concurrent colorectal carcinoma, compared to normal mucosa samples (Zhang et al., 2003a; Zhang et al., 2003b). Statistically, *REG4* mRNA was more expressed in colorectal cancers (especially mucinous carcinomas) than in normal colorectal mucosa (Violette et al., 2003). Combining these results, we conclude that up-regulated *REG4* mRNA expression is markedly observed in colorectal adenoma and adenocarcinoma.

At the protein level, REG4 expression was observed in both the middle and outer parts of crypts and superficial epithelium, especially goblets (Granlund et al., 2011). Statistically, REG4 expression was significantly lower in colorectal cancer than in normal mucosa or adenomas, and inversely correlated with poor differentiation, venous invasion, low expression of MUC2 and EGFR phosphorylated at Tyr1068 (Li et al., 2010). REG4 expression was less frequently observed in colorectal cancer than in adjacent non-neoplastic mucosa, in well- and moderately-differentiated adenomas than in mucinous carcinoma (Zheng et al., 2011), and in the cancers of the right colon than in the left colon and rectum, respectively (Kang et al., 2021). Further study showed that REG4 expression was associated with lymph node metastasis, distant metastasis, metastatic recurrence in the liver, advanced TNM stage, histologic grade, and MMP-7 expression in colorectal cancer (Oue et al., 2007; Zhu et al., 2015). Oue et al. (2007) found that the preoperative serum REG4 concentration was not elevated in patients with colorectal cancer at stages 0–III, but was significantly elevated in those at stage IV. Additionally, REG4 expression, as an independent predictor, was significantly linked to a worse prognosis in patients with colorectal cancer (Oue et al., 2007; Numata et al., 2011; He et al., 2014). However, Kaprio et al. (2014) reported that REG4 expression was an independent marker of a lower risk of death for patients with non-mucinous colorectal cancer, 65 years and younger, within 5 years. These findings demonstrated that aberrant REG4 expression is involved in the colorectal adenoma–adenocarcinoma sequence, and can be used to indicate the aggressive behaviors and prognosis of colorectal cancers.

A body of evidence has shown that REG4 was markedly related to chemoresistance, migration, and invasion of cancer cells. Violette et al. (Zhang et al., 2003b) discovered that REG4 protein was strongly expressed in drug-resistant rectal cancer cells, but expressed weakly in drug-sensitive rectal cancer cells. REG4 expression was found to correlate with γ-radiation sensitivity in rectal cancer patients receiving radiotherapy (Kobunai et al., 2011). In radiochemotherapy (RCT)-sensitive colorectal cancer cells, REG4 expression was down-regulated, while it was increased in radiochemoresistant cells (Gao et al., 2021). REG4–overexpressing cells had a high survival rate and showed few DNA breaks after irradiation (He et al., 2014). rhREG4 significantly induced resistance to ionizing radiation in colon adenocarcinoma cells by promoting anti-apoptotic Bcl-xL and Bcl-2 expression (Numata et al., 2011). Additionally, rhREG4 stimulated cell growth in a paracrine manner. Notably, REG4 promoted migration and invasion of colorectal cancer cells via its carbohydrate-recognition domain in both autocrine and paracrine manners, which was significantly decreased by anti-REG4 antibody (Guo et al., 2010; Rafa et al., 2010). Nanakin et al. (2007) found that REG4 expression was stimulated by TNFα, epidermal growth factor (EGF), bFGF, and HGF in colon cancer cells, and then promoted cell proliferation and resistance to H_2_O_2_-induced apoptosis. These data indicated that REG4 might be identified as a potential marker for RCT resistance.

In an animal model, *REG4* mRNA was elevated in the intestine of APC^min/+^ mice carrying APC mutation at codon 850 for transcription stop before a spontaneous second mutation of APC. Adenomas from 14-week-old APC^min/+^ mice showed significantly up-regulated expression of Bcl-2 and REG4 (Bishnupuri et al., 2006a). Sorted REG4-positive deep crypt secretory (DCS) cells facilitated organoid formation of single Lgr5 (+) stem cells, and DCS cells overwhelmingly originated from Lgr5 (+) stem cells by both Notch inactivation and Wnt activation (Sasaki et al., 2016). In an organoid model, mutant *KRAS*-induced REG4 promoted colorectal cancer stemness via a Wnt/β-catenin pathway (Hwang et al., 2020). This was also evidenced by the positive correlation of cancer stem markers with REG4 in intestinal tumors from APC^min+^/KrasG12D LA2 mice. These results indicated that REG4 might be involved in the colorectal adenoma–adenocarcinoma sequence by the regulation of local stem cells.

### Pancreatic cancer

In pancreatic tumors, *REG4* mRNA expression was significantly higher in intestinal-type rather than in gastric-type intraductal papillary mucinous neoplasms and normal pancreatic ductal epithelium. REG4 expression was higher in borderline lesions and carcinoma than in adenoma, and in colloid carcinoma than that in tubular carcinoma, respectively, and positively correlated with CDX2 expression (Nakata et al., 2009). A high serum REG4 level could be used to discriminate chronic pancreatitis and pancreatic ductal adenocarcinoma, as well as predict worse survival (Takehara et al., 2006; Takayama et al., 2010; Saukkonen et al., 2018). The pancreatic cancer patients with a higher serum REG4 concentration had an unfavorable response to RCT and frequently experienced local recurrence after surgery (Eguchi et al., 2009). The knockdown of REG4 or an anti-REG4 antibody both attenuated the cell viability of pancreatic cancer cells, while rhREG4 exposure showed the opposite effect in a dose-dependent manner (Eguchi et al., 2009). He et al. (2012) found that REG4 promoted not only tumor growth but also invasion of pancreatic cancer cells by up-regulating MMP-7 and MMP-9. REG4–overexpressing pancreatic cancer cells were resistant to gemcitabine and γ-radiation (Eguchi et al., 2009). These data suggested that REG4 overexpression contributed to pancreatic carcinogenesis and subsequent progression by promoting proliferation, invasion, and RCT resistance.

### Gallbladder cancer

*REG4* mRNA expression was significantly higher in gallbladder adenocarcinoma than peritumoral normal tissues, adenoma, and cholecystitis (Yang et al., 2016), in line with findings by Tamura et al. (Tamura et al., 2009). Immunohistochemically, REG4 was negative in all normal gallbladders and cholelithiasis, but positive in 50% of intestinal metaplasia with adenomyomatosis, and positive in 56% of gallbladder carcinomas (Tamura et al., 2009). REG4 expression was positively correlated with dedifferentiation, local invasiveness, and lymph node metastasis of gallbladder cancer. REG4 expression was independently associated with a poor prognosis in patients with advanced gallbladder cancer (Yang et al., 2016). A high serum REG4 level was evident preoperatively in four (33%) of 12 patients with gallbladder cancer, but not in benign diseases, and was postoperatively reduced (Tamura et al., 2009). These findings indicated that REG4 is involved in the carcinogenesis and subsequent progression of gallbladder adenocarcinoma.

### Ovarian cancer

In ovarian carcinogenesis, *REG4* mRNA and protein levels were higher in ovarian tumors than in normal ovaries, in mucinous carcinomas than in serous carcinomas, and in well- and moderately-differentiated carcinomas than in poorly-differentiated carcinomas, respectively (Chen et al., 2015), in line with another report (Xiang et al., 2022). REG4 protein expression was significantly higher in ovarian mucinous borderline tumors and mucinous carcinomas than in mucinous cystadenomas, and was more closely associated with ovarian borderline, intestinal-type, mucinous tumors rather than in endocervical-like type tumors. A significant positive correlation existed between CDX2 and REG4 expression in primary ovarian mucinous tumors (Huang et al., 2014b). High *REG4* mRNA expression was inversely associated with inferior overall, progression-free and post-progression survival in patients with ovarian cancer receiving platinum chemotherapy (Xiang et al., 2022). As an independent factor, the expression of REG4 was an overall or relapse-free poor prognostic factor for patients with ovarian cancer (Chen et al., 2015). In ovarian cancer cells, REG4 expression or 5-FU chemoresistance was enhanced by CDX2 transfection, in contrast to CDX2 knockdown (Koh et al., 2019). Either REG4 overexpression or rhREG4 treatment promoted proliferation, G_2_/S progression, anti-apoptosis, migration, invasion, and cisplatin and paclitaxel resistance in ovarian cancer cells (Chen et al., 2015; Xiang et al., 2022). Taken together, REG4 overexpression might play an important role in ovarian carcinogenesis and subsequent aggressiveness.

### Urothelial cancer

In renal clear cell carcinoma, the immunoexpression of REG4 was not detectable due to a lack of neuroendocrine and intestinal differentiation (Hayashi et al., 2009). In prostate cancer, 14 (14%) of 98 cases had tissues positive for REG4 staining, which was associated with MUC2 and chromogranin A expression. The expression of REG4 was a significant prognostic factor and independent predictor of the relapse-free survival of patients with prostate cancer. In prostate cancer, the serum REG4 concentration was significantly higher in patients with prostate cancer than in control individuals (Ohara et al., 2008). The overexpression of REG4 was also observed in hormone refractory xenografts and refractory metastatic prostate cancer (Gu et al., 2005). rhREG4 treatment enhanced EGFR phosphorylation in prostate cancer cells (Ohara et al., 2008). Taking these findings together, we speculated that REG4 was involved in prostate carcinogenesis and subsequent progression, especially in those patients showing intestinal mucin and neuroendocrine differentiation.

### Neuroendocrine tumors and SRCC

REG4 is physiologically found in selected enteroendocrine cells (EEC). REG4-positive gastric EECs were associated with serotonin, gastrin, somatostatin, and pancreatic polypeptide (Sentani et al., 2010). REG4 was differentially expressed in ECCs of the small intestine and colon (Heiskala and Andersson, 2013). REG4 showed a cellular co-distribution with serotonin, substance P or chromogranin A in the gastrointestinal tract. Subpopulations of REG4-positive cells overlapped with EECs containing GLP-1, GLP-2, peptide YY, secretin, and ghrelin, relying on the anatomical sites of the tissues. Therefore, REG4 is thought to be involved in intestinal and neuroendocrine differentiation. Oue et al. (2005) found that insulin-secreting β cells of the pancreas were positive for REG4. Of 21 gastric SRCCs, 16 colorectal SRCCs, 10 breast SRCCs, and 47 lung SRCCs, all gastric and colorectal SRCCs showed REG4 immunopositivity, but the others did not indicate that REG4 might be a biomarker specific for gastrointestinal SRCCs (Sentani et al., 2008).

## Others

In the esophagus, REG4 staining was not detected in squamous cell carcinoma (SCC) and small cell carcinomas, whereas REG4 staining was found in four of 10 (40%) adenocarcinoma samples. The serum REG4 level was significantly higher in patients with SCC than in control participants, and correlated with the control participants’ age (Oue et al., 2011). In the lung, REG4 was highly expressed in *KRAS*-mutant adenocarcinoma with thyroid transcription factor-1 (TTF-1) hypoexpression. Silencing significantly REG4 reduced proliferation and tumor growth, and arrested the cell cycle by regulating E2F targets and the G_2_/M checkpoint (Sun et al., 2019). In glioma, REG4 expression was significantly higher in tumor than normal brain tissues. REG4 immunoreactivity was significantly associated with advanced pathological grade and a low Karnofsky performance score, and short survival as an independent prognostic factor (Wang et al., 2012). Sasahira et al. (2008) found that REG4 was expressed in salivary duct epithelia and acinus myoepithelia, but not in squamous epithelia. REG4 expression was found in 41% (17/41) of adenoid cystic carcinomas (ACCs), but not in SCCs, and was associated with lymph node involvement and a poor prognosis in ACC. These findings indicated that REG4 expression was not detectable in SCC, but in glioma, and ACC.

## Conclusion and perspectives

In conclusion, altered REG4 expression is involved in infection and inflammation, and in gastric, colorectal, pancreatic, gallbladder, ovarian and urothelial cancers, where it is closely linked to aggressive behaviors and a poor prognosis. REG4 expression and recombinant REG4 aggravated tumorigenesis, proliferation, anti-apoptosis, chemoradioresistance, migration, invasion, peritoneal dissemination, tumor growth, and cancer stemness by EGFR/Akt/AP-1 and Akt/GSK3β/β-catenin/TCF-4 pathways. Histologically, REG4 protein is highly expressed in neuroendocrine tumors and SRCCs. In accordance with recent findings about REG4, we believe that REG4 should be used as a biomarker for SRCC and neuroendocrine tumors, and contributes to the histogenesis of gastric intestinal–metaplasia–globoid dysplasia–SRCC. Aberrant REG4 expression should be employed to predict the tumorigenesis, aggressive behaviors and poor prognosis of malignancies, and the lavage REG4 level should be determined to guard against peritoneal dissemination.

## Glossary

ACCs: adenoid cystic carcinomas
AP-1: activator protein-1
ATF2: activating transcription factor 2
Bcl-xl: B cell lymphoma-extra large
Bcl-2: B cell lymphoma two
bFGF: basic fibroblast growth factor
CDX2: caudal type homeobox two
CD44ICD: CD44 intracytoplasmic domain
C-type lectin: calcium-dependent lectin
DCS: deep crypt secretory
EEC: enteroendocrine cells
EGF: epidermal growth factor
EGFR: epidermal growth factor receptor
GATA6: GATA binding protein six
GLI1: GLI family zinc finger one;
GPR37: G protein-coupled receptor 37
GSK3β: glycogen synthase kinase three β
HGF: hepatocyte growth factor
IL: interleukin
Klf4: Kruppel-like factor 4
Lgr5: leucine-rich repeat-containing G-protein coupled receptor five
miR: MicroRNA
MMP-7: matrix metalloproteinase-7
MUC2: mucin two
RCT: radiochemotherapy
REG: regenerating islet-derived
REG4: Regenerating islet-derived four
rhREG4: recombinant human REG4
SCC: squamous cell carcinoma
SOX2: SRY-box transcription factor 2
SOX9: SRY-box transcription factor 9
SP1: specificity protein one
SRCCs: signet ring cell carcinomas
TCF-4: transcription factor 4
TGF: transforming growth factor
TNF: tumor necrosis factor
TTF-1: transcription factor-1;
UC: ulcerative colitis
5-FU: 5-fluorouracil

## References

[B1] Azman J. Starcevic Klasan G. Ivanac D. Picard A. Jurisic-Erzen D. Nikolic M. (2011). Reg IV protein and mRNA expression in different rat organs. Acta Histochem. 113 (8), 793–797. 10.1016/j.acthis.2010.11.008 21168191

[B2] Bishnupuri K. S. Luo Q. Korzenik J. R. Henderson J. O. Houchen C. W. Anant S. (2006a). Dysregulation of Reg gene expression occurs early in gastrointestinal tumorigenesis and regulates anti-apoptotic genes. Cancer Biol. Ther. 5 (12), 1714–1720. 10.4161/cbt.5.12.3469 17106246

[B3] Bishnupuri K. S. Luo Q. Murmu N. Houchen C. W. Anant S. Dieckgraefe B. K. (2006b). Reg IV activates the epidermal growth factor receptor/Akt/AP-1 signaling pathway in colon adenocarcinomas. Gastroenterology 130 (1), 137–149. 10.1053/j.gastro.2005.10.001 16401477

[B4] Bishnupuri K. S. Luo Q. Sainathan S. K. Kikuchi K. Sureban S. M. Sabarinathan M. (2010). Reg IV regulates normal intestinal and colorectal cancer cell susceptibility to radiation-induced apoptosis. Gastroenterology 138 (2), 616–626. 10.1053/j.gastro.2009.10.050 19900450 PMC2819553

[B5] Bishnupuri K. S. Sainathan S. K. Bishnupuri K. Leahy D. R. Luo Q. Anant S. (2014). Reg4-induced mitogenesis involves Akt-GSK3β-β-Catenin-TCF-4 signaling in human colorectal cancer. Mol. Carcinog. 53 (101), E169–E180. 10.1002/mc.22088 24151146 PMC4565142

[B6] Bishnupuri K. S. Sainathan S. K. Ciorba M. A. Houchen C. W. Dieckgraefe B. K. (2022). Reg4 interacts with CD44 to regulate proliferation and stemness of colorectal and pancreatic cancer cells. Mol. Cancer Res. 20 (3), 387–399. 10.1158/1541-7786.MCR-21-0224 34753802

[B7] Chai D. Du H. Li K. Zhang X. Li X. Zhao X. (2021). CDX2 and Reg IV expression and correlation in gastric cancer. BMC Gastroenterol. 21 (1), 92. 10.1186/s12876-021-01678-9 33639844 PMC7913228

[B8] Chen S. Gou W. F. Zhao S. Niu Z. F. Zhao Y. Takano Y. (2015). The role of the REG4 gene and its encoding product in ovarian epithelial carcinoma. BMC Cancer 15, 471. 10.1186/s12885-015-1435-2 26077911 PMC4469329

[B9] Du F. Yao Z. W. (2013). The expression patterns of Reg IV gene in normal rat reproduction system. J. Exp. Zool. A Ecol. Genet. Physiol. 319 (1), 32–38. 10.1002/jez.1771 23203400

[B10] Duan Y. Hu L. Liu B. Yu B. Li J. Yan M. (2014). Tumor suppressor miR-24 restrains gastric cancer progression by downregulating RegIV. Mol. Cancer 13, 127. 10.1186/1476-4598-13-127 24886316 PMC4041902

[B11] Eguchi H. Ishikawa O. Ohigashi H. Takahashi H. Yano M. Nishiyama K. (2009). Serum REG4 level is a predictive biomarker for the response to preoperative chemoradiotherapy in patients with pancreatic cancer. Pancreas 38 (7), 791–798. 10.1097/MPA.0b013e3181ac5337 19546835

[B12] Gao L. Wu X. Zhang L. Dai Y. Zhu Z. Zhi Y. (2021). REG4 is a Potential biomarker for radiochemotherapy sensitivity in colorectal cancer. Onco. Targets. Ther. 14, 1605–1611. 10.2147/OTT.S296031 33688207 PMC7936684

[B13] Granlund A. V. Beisvag V. Torp S. H. Flatberg A. Kleveland P. M. Ostvik A. E. (2011). Activation of REG family proteins in colitis. Scand. J. Gastroenterol. 46 (11), 1316–1323. 10.3109/00365521.2011.605463 21992413 PMC3212911

[B14] Gu Z. Rubin M. A. Yang Y. Deprimo S. E. Zhao H. Horvath S. (2005). Reg IV: A promising marker of hormone refractory metastatic prostate cancer. Clin. Cancer Res. 11 (6), 2237–2243. 10.1158/1078-0432.CCR-04-0356 15788672

[B15] Guo Y. Xu J. Li N. Gao F. Huang P. (2010). RegIV potentiates colorectal carcinoma cell migration and invasion via its CRD domain. Cancer Genet. cytogenet. 199 (1), 38–44. 10.1016/j.cancergencyto.2010.01.011 20417867

[B16] Hartupee J. C. Zhang H. Bonaldo M. F. Soares M. B. Dieckgraefe B. K. (2001). Isolation and characterization of a cDNA encoding a novel member of the human regenerating protein family: Reg IV. Biochim. Biophys. Acta 1518 (3), 287–293. 10.1016/s0167-4781(00)00284-0 11311942

[B17] Hayashi T. Matsubara A. Ohara S. Mita K. Hasegawa Y. Usui T. (2009). Immunohistochemical analysis of Reg IV in urogenital organs: Frequent expression of Reg IV in prostate cancer and potential utility as serum tumor marker. Oncol. Rep. 21 (1), 95–100. 19082448

[B18] He H. L. Lee Y. E. Shiue Y. L. Lee S. W. Lin L. C. Chen T. J. (2014). Overexpression of REG4 confers an independent negative prognosticator in rectal cancers receiving concurrent chemoradiotherapy. J. Surg. Oncol. 110 (8), 1002–1010. 10.1002/jso.23764 25155043

[B19] He X. J. Jiang X. T. Ma Y. Y. Xia Y. J. Wang H. J. Guan T. P. (2012). REG4 contributes to the invasiveness of pancreatic cancer by upregulating MMP-7 and MMP-9. Cancer Sci. 103 (12), 2082–2091. 10.1111/cas.12018 22957785 PMC7659226

[B20] Heiskala K. Andersson L. C. (2013). Reg IV is differently expressed in enteroendocrine cells of human small intestine and colon. Regul. Pept. 183, 27–34. 10.1016/j.regpep.2013.03.007 23499801

[B21] Ho M. R. Lou Y. C. Wei S. Y. Luo S. C. Lin W. C. Lyu P. C. (2010). Human RegIV protein adopts a typical C-type lectin fold but binds mannan with two calcium-independent sites. J. Mol. Biol. 402 (4), 682–695. 10.1016/j.jmb.2010.07.061 20692269

[B22] Hu G. Shen J. Cheng L. Guo C. Xu X. Wang F. (2011). Reg4 protects against acinar cell necrosis in experimental pancreatitis. Gut 60 (6), 820–828. 10.1136/gut.2010.215178 21193457

[B23] Huang J. Yang Y. Yang J. Li X. (2014a). Regenerating gene family member 4 promotes growth and migration of gastric cancer through protein kinase B pathway. Int. J. Clin. Exp. Med. 7 (9), 3037–3044. 25356179 PMC4211829

[B24] Huang Q. Chen X. Lu W. Lai M. Lu B. (2014b). Expression of REG4 in ovarian mucinous tumors. Appl. Immunohistochem. Mol. Morphol. 22 (4), 295–301. 10.1097/PAI.0b013e3182936d8e 23958547

[B25] Hwang J. H. Yoon J. Cho Y. H. Cha P. H. Park J. C. Choi K. Y. (2020). A mutant KRAS-induced factor REG4 promotes cancer stem cell properties via Wnt/β-catenin signaling. Int. J. Cancer 146 (10), 2877–2890. 10.1002/ijc.32728 31605540

[B26] Jin J. Lv H. Wu J. Li D. Chen K. Zhang F. (2017). Regenerating family member 4 (Reg4) enhances 5-Fluorouracil resistance of gastric cancer through activating MAPK/Erk/Bim signaling pathway. Med. Sci. Monit. 23, 3715–3721. 10.12659/msm.903134 28759561 PMC5549713

[B27] Kang G. Oh I. Pyo J. Kang D. Son B. (2021). Clinicopathological significance and prognostic implications of REG4 immunohistochemical expression in colorectal cancer. Med. Kaunas. 57 (9), 938. 10.3390/medicina57090938 PMC8464993 34577861

[B28] Kaprio T. Hagström J. Mustonen H. Koskensalo S. Andersson L. C. Haglund C. (2014). REG4 independently predicts better prognosis in non-mucinous colorectal cancer. PLoS One 9 (10), e109600. 10.1371/journal.pone.0109600 25295732 PMC4190354

[B29] Katsuno Y. Ehata S. Yashiro M. Yanagihara K. Hirakawa K. Miyazono K. (2012). Coordinated expression of REG4 and aldehyde dehydrogenase 1 regulating tumourigenic capacity of diffuse-type gastric carcinoma-initiating cells is inhibited by TGF-β. J. Pathol. 228 (3), 391–404. 10.1002/path.4020 22430847

[B30] Kawasaki Y. Matsumura K. Miyamoto M. Tsuji S. Okuno M. Suda S. (2015). REG4 is a transcriptional target of GATA6 and is essential for colorectal tumorigenesis. Sci. Rep. 5, 14291. 10.1038/srep14291 26387746 PMC4585703

[B31] Kobayashi Y. Niwa Y. Tajika M. Kawai H. Kondo S. Hara K. (2010). Serum tumor antigen REG4 as a useful diagnostic biomarker in gastric cancer. Hepatogastroenterology. 57 (104), 1631–1634. 21443133

[B32] Kobunai T. Watanabe T. Fukusato T. (2011). REG4, NEIL2, and BIRC5 gene expression correlates with gamma-radiation sensitivity in patients with rectal cancer receiving radiotherapy. Anticancer Res. 31 (12), 4147–4153. 22199273

[B33] Koh I. Nosaka S. Sekine M. Sugimoto J. Hirata E. Kudo Y. (2019). Regulation of REG4 expression and prediction of 5-Fluorouracil sensitivity by CDX2 in ovarian mucinous carcinoma. Cancer Genomics Proteomics 16 (6), 481–490. 10.21873/cgp.20151 31659102 PMC6885360

[B34] Kuniyasu H. Oue N. Sasahira T. Yi L. Moriwaka Y. Shimomoto T. (2009). Reg IV enhances peritoneal metastasis in gastric carcinomas. Cell Prolif. 42 (1), 110–121. 10.1111/j.1365-2184.2008.00577.x 19143768 PMC6495736

[B35] Li X. H. Zheng Y. Zheng H. C. Takahashi H. Yang X. H. Masuda S. (2010). REG IV overexpression in an early stage of colorectal carcinogenesis: An immunohistochemical study. Histol. Histopathol. 25 (4), 473–484. 10.14670/HH-25.473 20183800

[B36] Li X. J. Zhu F. Li B. Zhang D. Liang C. W. (2021). Recombinant human regenerating gene 4 attenuates the severity of osteoarthritis by promoting the proliferation of articular chondrocyte in an animal model. Curr. Mol. Pharmacol. 15, 693–699. 10.2174/1874467214666210901163144 34488597

[B37] Li Y. G. Ji D. F. Zhong S. Zhu J. X. Chen S. Hu G. Y. (2011). Anti-tumor effects of proteoglycan from Phellinus linteus by immunomodulating and inhibiting Reg IV/EGFR/Akt signaling pathway in colorectal carcinoma. Int. J. Biol. Macromol. 48 (3), 511–517. 10.1016/j.ijbiomac.2011.01.014 21262260

[B38] Liu C. M. Hsieh C. L. He Y. C. Lo S. J. Liang J. A. Hsieh T. F. (2013). *In vivo* targeting of ADAM9 gene expression using lentivirus-delivered shRNA suppresses prostate cancer growth by regulating REG4 dependent cell cycle progression. PLoS One 8 (1), e53795. 10.1371/journal.pone.0053795 23342005 PMC3547060

[B39] Lu S. Bevier M. Huhn S. Sainz J. Lascorz J. Pardini B. (2013). Genetic variants in C-type lectin genes are associated with colorectal cancer susceptibility and clinical outcome. Int. J. Cancer 133 (10), 2325–2333. 10.1002/ijc.28251 23650115

[B40] Ma X. Wu D. Zhou S. Wan F. Liu H. Xu X. (2016). The pancreatic cancer secreted REG4 promotes macrophage polarization to M2 through EGFR/AKT/CREB pathway. Oncol. Rep. 35 (1), 189–196. 10.3892/or.2015.4357 26531138

[B41] Mitani Y. Oue N. Matsumura S. Yoshida K. Noguchi T. Ito M. (2007). Reg IV is a serum biomarker for gastric cancer patients and predicts response to 5-fluorouracil-based chemotherapy. Oncogene 26 (30), 4383–4393. 10.1038/sj.onc.1210215 17237819

[B42] Miyagawa K. Sakakura C. Nakashima S. Yoshikawa T. Fukuda K. Kin S. (2008). Overexpression of RegIV in peritoneal dissemination of gastric cancer and its potential as A novel marker for the detection of peritoneal micrometastasis. Anticancer Res. 28 (2B), 1169–1179. 18505053

[B43] Moon J. H. Fujiwara Y. Nakamura Y. Okada K. Hanada H. Sakakura C. (2012). REGIV as a potential biomarker for peritoneal dissemination in gastric adenocarcinoma. J. Surg. Oncol. 105 (2), 189–194. 10.1002/jso.22021 21780125

[B44] Naito Y. Oue N. Hinoi T. Sakamoto N. Sentani K. Ohdan H. (2012). Reg IV is a direct target of intestinal transcriptional factor CDX2 in gastric cancer. PLoS One 7 (11), e47545. 10.1371/journal.pone.0047545 23133598 PMC3487720

[B45] Nakata K. Nagai E. Ohuchida K. Aishima S. Hayashi A. Miyasaka Y. (2009). REG4 is associated with carcinogenesis in the 'intestinal' pathway of intraductal papillary mucinous neoplasms. Mod. Pathol. 22 (3), 460–468. 10.1038/modpathol.2008.205 19136934

[B46] Nanakin A. Fukui H. Fujii S. Sekikawa A. Kanda N. Hisatsune H. (2007). Expression of the REG IV gene in ulcerative colitis. Lab. Invest. 87 (3), 304–314. 10.1038/labinvest.3700507 17260007

[B47] Nata K. Liu Y. Xu L. Ikeda T. Akiyama T. Noguchi N. (2004). Molecular cloning, expression and chromosomal localization of a novel human REG family gene, REG III. Gene 340 (1), 161–170. 10.1016/j.gene.2004.06.010 15556304

[B48] Numata M. Oshima T. Yoshihara K. Watanabe T. Tsuchida K. Tamagawa H. (2011). Relationship between RegIV gene expression to outcomes in colorectal cancer. J. Surg. Oncol. 104 (2), 205–209. 10.1002/jso.21906 21381041

[B49] Ohara S. Oue N. Matsubara A. Mita K. Hasegawa Y. Hayashi T. (2008). Reg IV is an independent prognostic factor for relapse in patients with clinically localized prostate cancer. Cancer Sci. 99 (8), 1570–1577. 10.1111/j.1349-7006.2008.00846.x 18754868 PMC11158611

[B50] Oue N. Kuniyasu H. Noguchi T. Sentani K. Ito M. Tanaka S. (2007). Serum concentration of reg IV in patients with colorectal cancer: Overexpression and high serum levels of reg IV are associated with liver metastasis. Oncology 72 (5-6), 371–380. 10.1159/000113147 18187959

[B51] Oue N. Mitani Y. Aung P. P. Sakakura C. Takeshima Y. Kaneko M. (2005). Expression and localization of Reg IV in human neoplastic and non-neoplastic tissues: Reg IV expression is associated with intestinal and neuroendocrine differentiation in gastric adenocarcinoma. J. Pathol. 207 (2), 185–198. 10.1002/path.1827 16086444

[B52] Oue N. Noguchi T. Anami K. Sentani K. Sakamoto N. Uraoka N. (2011). Serum concentration and expression of Reg IV in patients with esophageal cancer: Age-related elevation of serum Reg IV concentration. Oncol. Lett. 2 (2), 235–239. 10.3892/ol.2011.239 22866070 PMC3410574

[B53] Qi H. Wei J. Gao Y. Yang Y. Li Y. Zhu H. (2020). Reg4 and complement factor D prevent the overgrowth of *E. coli* in the mouse gut. Commun. Biol. 3 (1), 483. 10.1038/s42003-020-01219-2 32879431 PMC7468294

[B54] Rafa L. Dessein A. F. Devisme L. Buob D. Truant S. Porchet N. (2010). REG4 acts as a mitogenic, motility and pro-invasive factor for colon cancer cells. Int. J. Oncol. 36 (3), 689–698. 10.3892/ijo_00000544 20126989

[B55] Sasahira T. Oue N. Kirita T. Luo Y. Bhawal U. K. Fujii K. (2008). Reg IV expression is associated with cell growth and prognosis of adenoid cystic carcinoma in the salivary gland. Histopathology 53 (6), 667–675. 10.1111/j.1365-2559.2008.03188.x 19076683

[B56] Sasaki N. Sachs N. Wiebrands K. Ellenbroek S. I. Fumagalli A. Lyubimova A. (2016). Reg4+ deep crypt secretory cells function as epithelial niche for Lgr5+ stem cells in colon. Proc. Natl. Acad. Sci. U. S. A. 113 (37), E5399–E5407. 10.1073/pnas.1607327113 27573849 PMC5027439

[B57] Saukkonen K. Hagström J. Mustonen H. Lehtinen L. Carpen O. Andersson L. C. (2018). Prognostic and diagnostic value of REG4 serum and tissue expression in pancreatic ductal adenocarcinoma. Tumour Biol. 40 (3), 1010428318761494. 10.1177/1010428318761494 29542402

[B58] Sentani K. Oue N. Noguchi T. Sakamoto N. Matsusaki K. Yasui W. (2010). Immunostaining of gastric cancer with neuroendocrine differentiation: Reg IV-positive neuroendocrine cells are associated with gastrin, serotonin, pancreatic polypeptide and somatostatin. Pathol. Int. 60 (4), 291–297. 10.1111/j.1440-1827.2010.02519.x 20403031

[B59] Sentani K. Oue N. Tashiro T. Sakamoto N. Nishisaka T. Fukuhara T. (2008). Immunohistochemical staining of Reg IV and claudin-18 is useful in the diagnosis of gastrointestinal signet ring cell carcinoma. Am. J. Surg. Pathol. 32 (8), 1182–1189. 10.1097/PAS.0b013e318163a8f8 18580680

[B60] Sninsky J. A. Bishnupuri K. S. González I. Trikalinos N. A. Chen L. Dieckgraefe B. K. (2021). Reg4 and its downstream transcriptional activator CD44ICD in stage II and III colorectal cancer. Oncotarget 12 (4), 278–291. 10.18632/oncotarget.27896 33659040 PMC7899555

[B61] Sun S. Hu Z. Huang S. Ye X. Wang J. Chang J. (2019). REG4 is an indicator for KRAS mutant lung adenocarcinoma with TTF-1 low expression. J. Cancer Res. Clin. Oncol. 145 (9), 2273–2283. 10.1007/s00432-019-02988-y 31428934 PMC11810320

[B62] Takasawa S. Tsuchida C. Sakuramoto-Tsuchida S. Takeda M. Itaya-Hironaka A. Yamauchi A. (2018). Expression of human REG family genes in inflammatory bowel disease and their molecular mechanism. Immunol. Res. 66 (6), 800–805. 10.1007/s12026-019-9067-2 30694514

[B63] Takayama R. Nakagawa H. Sawaki A. Mizuno N. Kawai H. Tajika M. (2010). Serum tumor antigen REG4 as a diagnostic biomarker in pancreatic ductal adenocarcinoma. J. Gastroenterol. 45 (1), 52–59. 10.1007/s00535-009-0114-y 19789838

[B64] Takehara A. Eguchi H. Ohigashi H. Ishikawa O. Kasugai T. Hosokawa M. (2006). Novel tumor marker REG4 detected in serum of patients with resectable pancreatic cancer and feasibility for antibody therapy targeting REG4. Cancer Sci. 97 (11), 1191–1197. 10.1111/j.1349-7006.2006.00297.x 16918991 PMC11159249

[B65] Tamura H. Ohtsuka M. Washiro M. Kimura F. Shimizu H. Yoshidome H. (2009). Reg IV expression and clinicopathologic features of gallbladder carcinoma. Hum. Pathol. 40 (12), 1686–1692. 10.1016/j.humpath.2009.06.001 19716164

[B66] Tao H. Q. He X. J. Ma Y. Y. Wang H. J. Xia Y. J. Ye Z. Y. (2011). Evaluation of REG4 for early diagnosis and prognosis of gastric cancer. Hum. Pathol. 42 (10), 1401–1409. 10.1016/j.humpath.2010.08.023 21419474

[B67] Violette S. Festor E. Pandrea-Vasile I. Mitchell V. Adida C. Dussaulx E. (2003). Reg IV, a new member of the regenerating gene family, is overexpressed in colorectal carcinomas. Int. J. Cancer 103 (2), 185–193. 10.1002/ijc.10788 12455032

[B68] Wang F. Xu L. Guo C. Ke A. Hu G. Xu X. (2011). Identification of RegIV as a novel GLI1 target gene in human pancreatic cancer. PLoS One 6 (4), e18434. 10.1371/journal.pone.0018434 21494603 PMC3073946

[B69] Wang H. Hu L. Zang M. Zhang B. Duan Y. Fan Z. (2016). REG4 promotes peritoneal metastasis of gastric cancer through GPR37. Oncotarget 7 (19), 27874–27888. 10.18632/oncotarget.8442 27036049 PMC5053694

[B70] Wang Q. Deng J. Yuan J. Wang L. Zhao Z. He S. (2012). Oncogenic Reg IV is a novel prognostic marker for glioma patient survival. Diagn. Pathol. 7, 69. 10.1186/1746-1596-7-69 22713481 PMC3465175

[B71] Xiang L. W. Xue H. Ha M. W. Yu D. Y. Xiao L. J. Zheng H. (2022). The effects of REG4 expression on chemoresistance of ovarian cancer. J. Obstet. Gynaecol. Tokyo. 1995., 1–9. 10.1080/01443615.2022.2106834 35929918

[B72] Xiao Y. Lu Y. Wang Y. Yan W. Cai W. (2019). Deficiency in intestinal epithelial Reg4 ameliorates intestinal inflammation and alters the colonic bacterial composition. Mucosal Immunol. 12 (4), 919–929. 10.1038/s41385-019-0161-5 30953001 PMC7744279

[B73] Yamagishi H. Fukui H. Sekikawa A. Kono T. Fujii S. Ichikawa K. (2009). Expression profile of REG family proteins REG ialpha and REG IV in advanced gastric cancer: Comparison with mucin phenotype and prognostic markers. Mod. Pathol. 22 (7), 906–913. 10.1038/modpathol.2009.41 19329938

[B74] Yang L. Lan S. Liu J. Yang Z. (2016). Expression of MK-1 and RegIV and its clinicopathological significances in the benign and malignant lesions of gallbladder. Diagn. Pathol. 6, 100. 10.1186/1746-1596-6-100 PMC3225305 22018336

[B75] Ying L. S. Yu J. L. Lu X. X. Ling Z. Q. (2013). Enhanced RegIV expression predicts the intrinsic 5-fluorouracil (5-FU) resistance in advanced gastric cancer. Dig. Dis. Sci. 58 (2), 414–422. 10.1007/s10620-012-2381-3 23010741

[B76] Yonemura Y. Takashima T. Miwa K. Miyazaki I. Yamamoto H. Okamoto H. (1984). Amelioration of diabetes mellitus in partially depancreatized rats by poly(ADP-ribose) synthetase inhibitors. Evidence of islet B-cell regeneration. Diabetes 33 (4), 401–404. 10.2337/diab.33.4.401 6323238

[B77] Zhang H. B. Luo H. C. Xin X. J. Zeng A. Z. (2015). Up-regulated Reg proteins induced by Interleukin-22 treatment ameliorate acute liver injury in rat model. Int. J. Clin. Exp. Med. 8 (1), 1253–1258. 25785121 PMC4358576

[B78] Zhang J. Zhu Z. Miao Z. Huang X. Sun Z. Xu H. (2021). The clinical significance and mechanisms of REG4 in human cancers. Front. Oncol. 10, 559230. 10.3389/fonc.2020.559230 33489872 PMC7819868

[B79] Zhang N. Chai D. Du H. Li K. Xie W. Li X. (2018). Expression of Reg IV and SOX9 and their correlation in human gastric cancer. BMC Cancer 18, 344. 10.1186/s12885-018-4285-x 29587675 PMC5870489

[B80] Zhang X. Q. Yu L. T. Du P. Yin T. Q. Zhang Z. Y. Xu Y. (2019). Single-chain antibody against Reg4 suppresses gastric cancer cell growth and enhances 5-FU-induced cell death *in vitro* . Anticancer. Agents Med. Chem. 19 (5), 610–619. 10.2174/1871520619666181122104720 30465515

[B81] Zhang Y. Lai M. Gu X. Luo M. Shao L. (2003). Reg IV, a differentially expressed gene in colorectal adenoma. Chin. Med. J. 116 (6), 918–922. 12877807

[B82] Zhang Y. Lai M. Lv B. Gu X. Wang H. Zhu Y. (2003). Overexpression of Reg IV in colorectal adenoma. Cancer Lett. 200 (1), 69–76. 10.1016/s0304-3835(03)00460-9 14550954

[B83] Zheng H. C. Sugawara A. Okamoto H. Takasawa S. Takahashi H. Masuda S. (2011). Expression profile of the REG gene family in colorectal carcinoma. J. Histochem. Cytochem. 59 (1), 106–115. 10.1369/jhc.2010.956961 21339177 PMC3201118

[B84] Zheng H. C. Xu X. Y. Yu M. Takahashi H. Masuda S. Takano Y. (2010). The role of Reg IV gene and its encoding product in gastric carcinogenesis. Hum. Pathol. 41 (1), 59–69. 10.1016/j.humpath.2009.06.013 19740514

[B85] Zhou W. Sun M. Wang D. L. Wang Y. Jin F. Zhang Y. Y. (2013). Silencing of RegIV by shRNA causes the loss of stemness properties of cancer stem cells in MKN45 gastric cancer cells. Oncol. Rep. 30 (6), 2685–2690. 10.3892/or.2013.2745 24064664

[B86] Zhu X. Han Y. Yuan C. Tu W. Qiu G. Lu S. (2015). Overexpression of Reg4, alone or combined with MMP-7 overexpression, is predictive of poor prognosis in colorectal cancer. Oncol. Rep. 33 (1), 320–328. 10.3892/or.2014.3559 25338725

